# The utilisation of health centre management by tuberculosis team in the effort to increase case finding

**DOI:** 10.1186/1471-2458-14-S1-O11

**Published:** 2014-01-29

**Authors:** Rahmat Bakhtiar, Hari Kusnanto, Barmawi Hisyam

**Affiliations:** 1 Faculty of Medicine, Mulawarman University, Samarinda East Kalimantan, Indonesia; 2 Universitas Gadjah Mada, Yogyakarta 55281, Indonesia

## Background

Case finding is a major problem in the programme of tuberculosis (TB) control at Kalimantan Timur. Low finding of new cases indicates that TB infection in the community is still relatively high and the programme is not yet focused on efforts to cut infection chain. Health centres with DOTS strategy are operated by standardised trained health staff known as health centre TB team. The problem is whether low case finding is related to the performance of TB team. The objective of the study was to identify potential problem facing TB team in the process of case finding based on management principles in the health centre.

## Materials and methods

The study used semi-qualitative design and was conducted in June-September 2011. Respondents were TB team health centres and head of health centre comprising as many as 48 individuals. There were 12 health centres with DOTS in three districts, i.e. Kutai Kartanegara, Kutai Barat and Kutai Timur. Selection of location was made with simple random sampling based on health centres consistently undertaking DOTS. Data were obtained through in-depth interviews with TB team and head of health centres.

## Results

TB team had not utilised the management tools of health centres in the effort to solve the problem of low TB case finding. Monthly mini workshops had not been utilised as media for information reference related to TB suspect identification in the field or tracing of drop out cases. Evaluation of recording cards was not well performed since it was only used as administrative requirement of the programme, not as material to analyse progress of the programme. Evaluation and monitoring had not been well practised by head of health centres so that information on TB case finding problem was limited to TB staff. District supervisors had not played the role as coach in tackling the existing problem. They seemed to prioritise on administrative problem of the programme Global Fund Project. Compliance of staff with finding algorithm was inconsistent. Despite its status as priority programme, TB programme activities were not well accommodated through case operational cost.

## Conclusions

The utilisation of health centre management in the effort to increase coverage of TB case finding in health centres was not yet optimum.

